# Immediate effects of scoliosis-specific corrective exercises on the Cobb angle after one week and after one year of practice

**DOI:** 10.1186/s13013-016-0101-z

**Published:** 2016-10-17

**Authors:** Karina Zapata, Eric C. Parent, Dan Sucato

**Affiliations:** 1 Therapy services, Texas Scottish Rite Hospital for Children, Dallas, TX 75219 USA; 2 Physical therapy, University of Alberta, Edmonton, AB T6G 2G4 USA; 3 Orthopaedics, Texas Scottish Rite Hospital for Children, Dallas, TX 75219 USA

## Abstract

**Background:**

We are unaware of any studies describing the immediate effects of scoliosis-specific exercises on the Cobb angle measured by radiograph. This study aimed to describe the differences between radiographs obtained with and without corrective exercises after initial training and after one year.

**Methods:**

A female with adolescent idiopathic scoliosis was first seen at age 13 years, 0 months with a Risser 0. She had a 43^o^ left lumbar, 15^o^ right thoracic curve. She was seen again after 6, 18 and 30 months and performed exercises from 18 to 30 months. She performed Barcelona Scoliosis Physical Therapy School (BSPTS) exercises for a four-curve type (lumbar dominant with pelvis deviation to the lumbar concave side). At 18 and 30 months, x-rays were obtained with and without performing corrective exercises.

**Results:**

At 6 months, her lumbar and thoracic curves measured 41^o^ and 28^o^, respectively. At 18 months, her lumbar and thoracic curves measured 47 ^o^ and 30^o^, respectively. Also at 18 months, immediately after her x-ray in the relaxed standing position, she performed her corrective exercises in standing with arms lowered for a second x-ray. Her lumbar and thoracic curves remained similar and measured 43^o^ and 32^o^, respectively. At 30 months, she performed unsolicited corrective exercises during the x-ray. Her lumbar and thoracic curves measured 26^o^ and 41^o^, respectively. Another x-ray in the relaxed position revealed lumbar and thoracic curves measuring 39^o^ and 35^o^, respectively. The immediate effect of corrective exercises after a year of training was a 33 % improvement at the lumbar spine compared to only a 9 % improvement the previous year.

**Conclusion:**

After initial training, corrective exercises during a standing x-ray did not significantly improve the Cobb angle for the major lumbar curve compared to the relaxed standing x-ray. However, a year after performing exercises, unsolicited corrective exercises resulted in a significantly improved Cobb angle compared to relaxed standing for the curve primarily targeted by the exercise program. Improved exercise ability and spinal flexibility may have contributed to the improved Cobb angle.

## Background

Auto-corrective exercises are integral to scoliosis-specific exercises [1–4]. The Cobb angle measured by radiograph is the most commonly used outcome measure to assess curve magnitude [5]. However, it is unclear whether and how corrective exercises affect the Cobb angle. A number of promising studies showed that Schroth corrective exercises for patients with scoliosis can slow curve progression or help patients achieve some curve correction [2, 3, 6–9]. We are also unaware of any studies describing the immediate effects of scoliosis-specific exercises on the Cobb angle. In an article on the Scientific Exercise Approach to Scoliosis (SEAS) method, Romano et al. [10] demonstrated x-ray images of one patient in a relaxed position and during corrective exercises [10]. However, no details were provided including the time frame of the x-rays and measurements of the exact Cobb angles. The aim of this study is to describe the differences between radiographs obtained with and without corrective exercises after initial training and after one year.

## Methods

Since this is a de-identified case study, IRB approval was not required.

## Case presentation

**Fig. 1:**
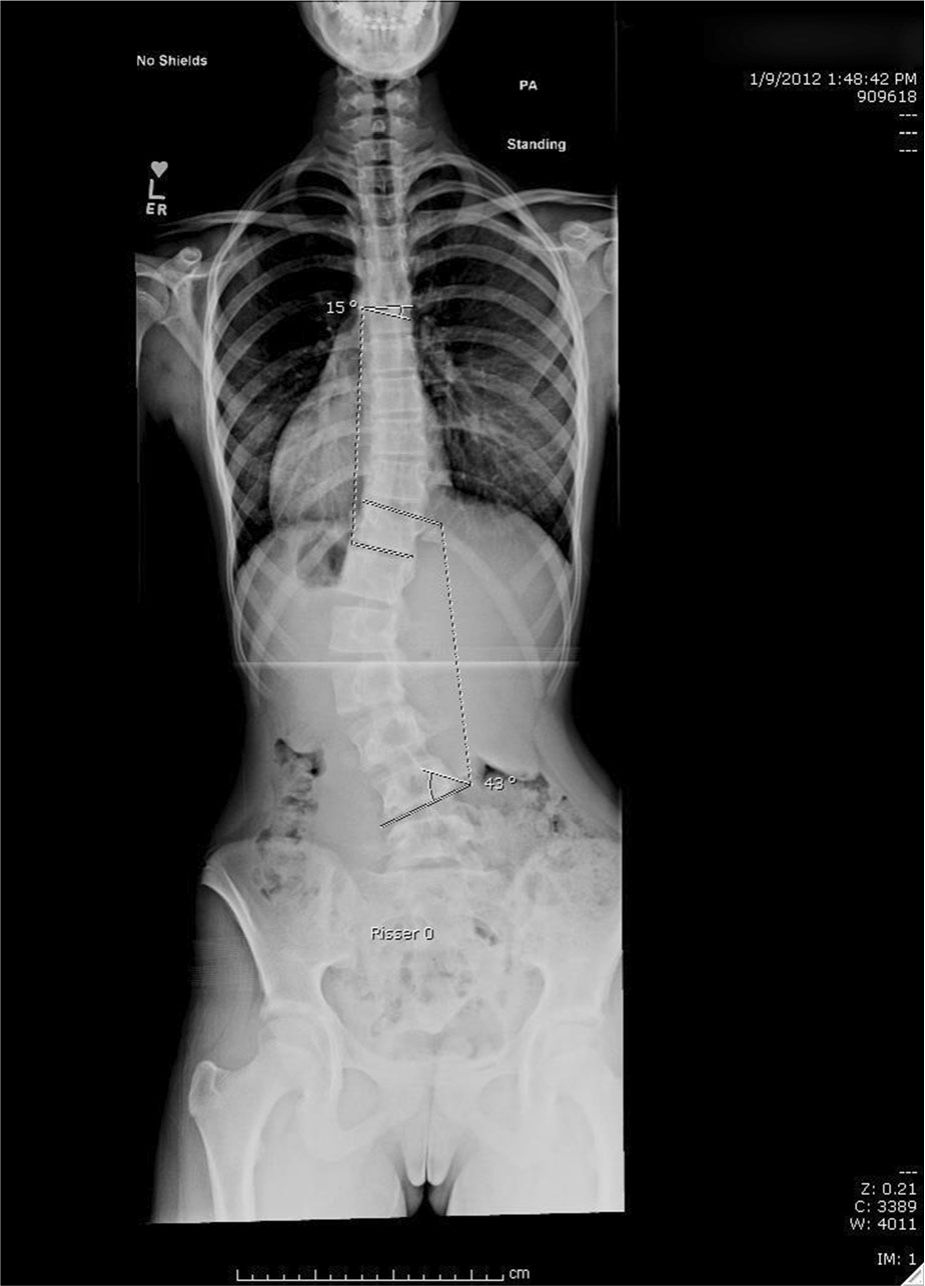
Baseline radiograph

**Fig. 2:**
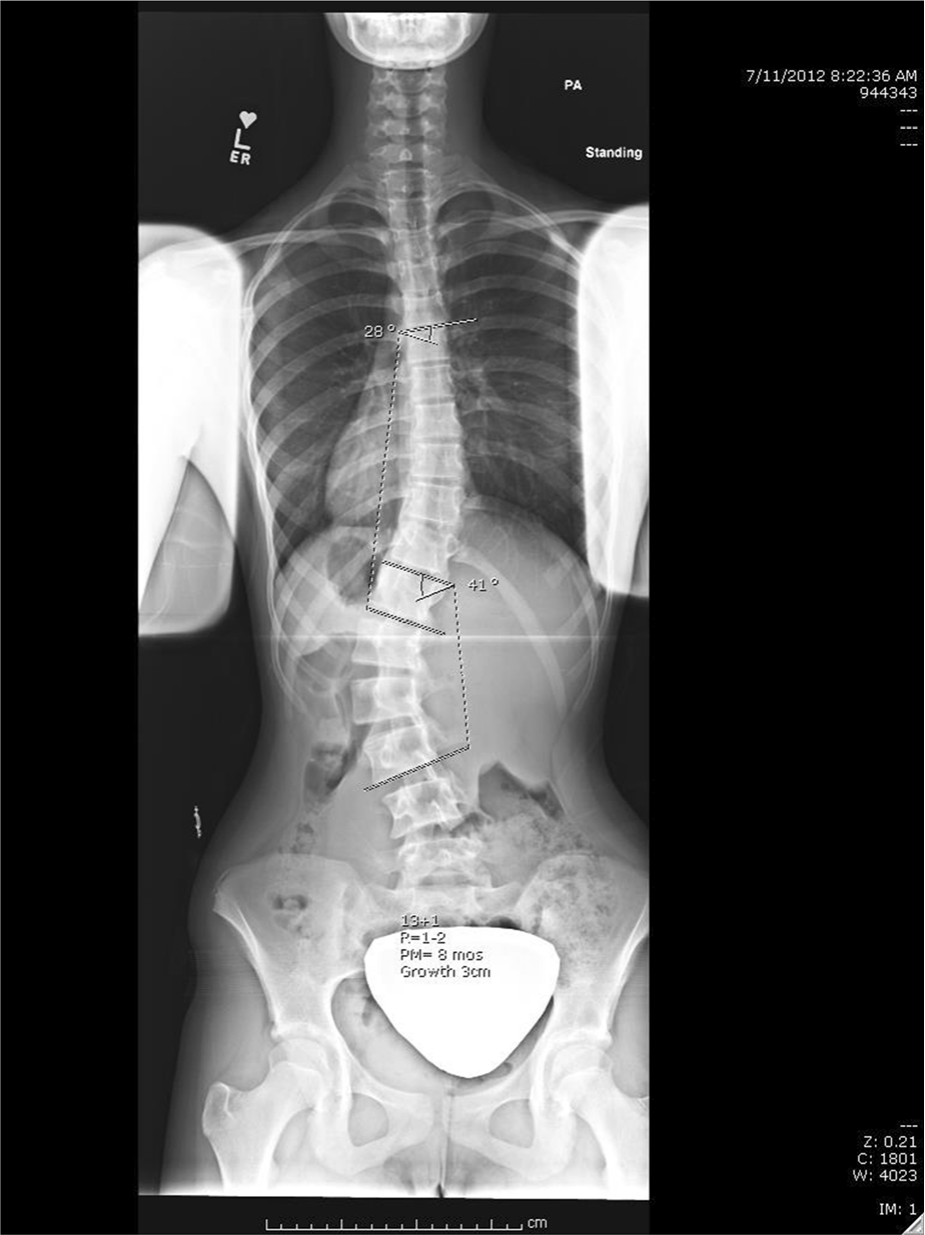
6 month visit

**Fig. 3:**
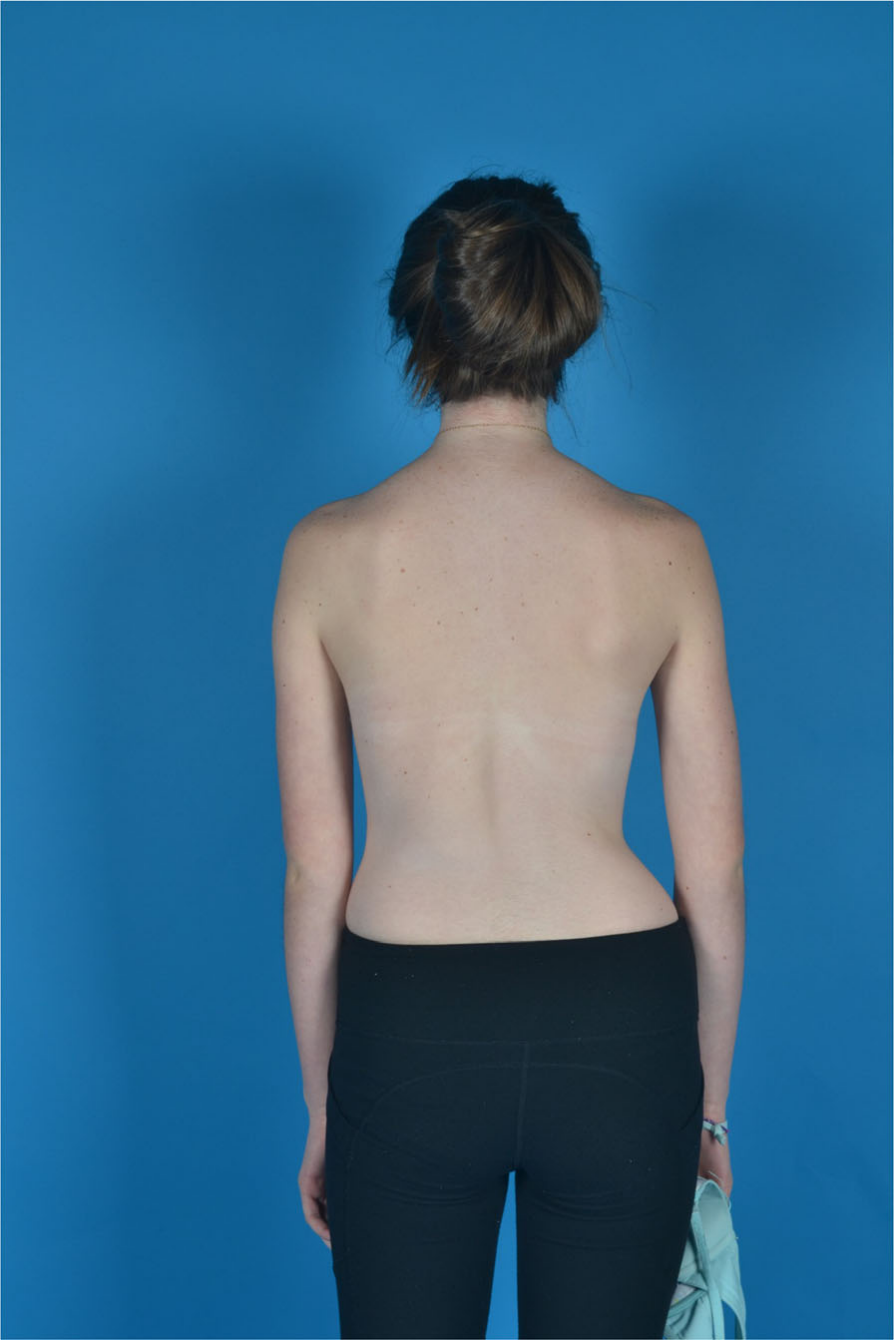
Clinical picture pre-exercise training at the 18 month visit

A female with adolescent idiopathic scoliosis was first seen at age 13, 0 months with a Risser 0, 2 months post-menarche. She had a 43^o^ left lumbar, 15^o^ right thoracic curve (Fig. 1). She had worn a Providence night-time brace for three months. At 6 months, her lumbar and thoracic curves measured 41^o^ and 28^o^, respectively. Her Risser was 1−2 and she had grown 3 cm (Fig. 2). She was seen again after 6, 18 and 30 months. At the 18 month visit, she was instructed in Barcelona Scoliosis Physical Therapy School (BSPTS) exercises for a four-curve type (lumbar dominant with pelvis deviation to the lumbar concave side) [11]. See Fig. 3 for a clinical picture of her relaxed standing posture. She attended 8 visits for 2 h each over one week of intensive instruction by a BSPTS-certified physical therapist with 7 months of experience. After discharge from physical therapy, she was asked to alternate performing 5 of 8 home exercises (semi-hanging, prone-on-knees, prone-on-stool, sidelying, side-sitting, rotational-sitting, sitting, standing) for 30 min per day, 5 times a week. No direct care was provided during this interval, since the patient did not have access to a trained physical therapist. She performed exercises from 18 to 30 months. At 18 and 30 months, x-rays were obtained with and without performing corrective exercises without the physical therapist present.

## Results

**Fig. 4:**
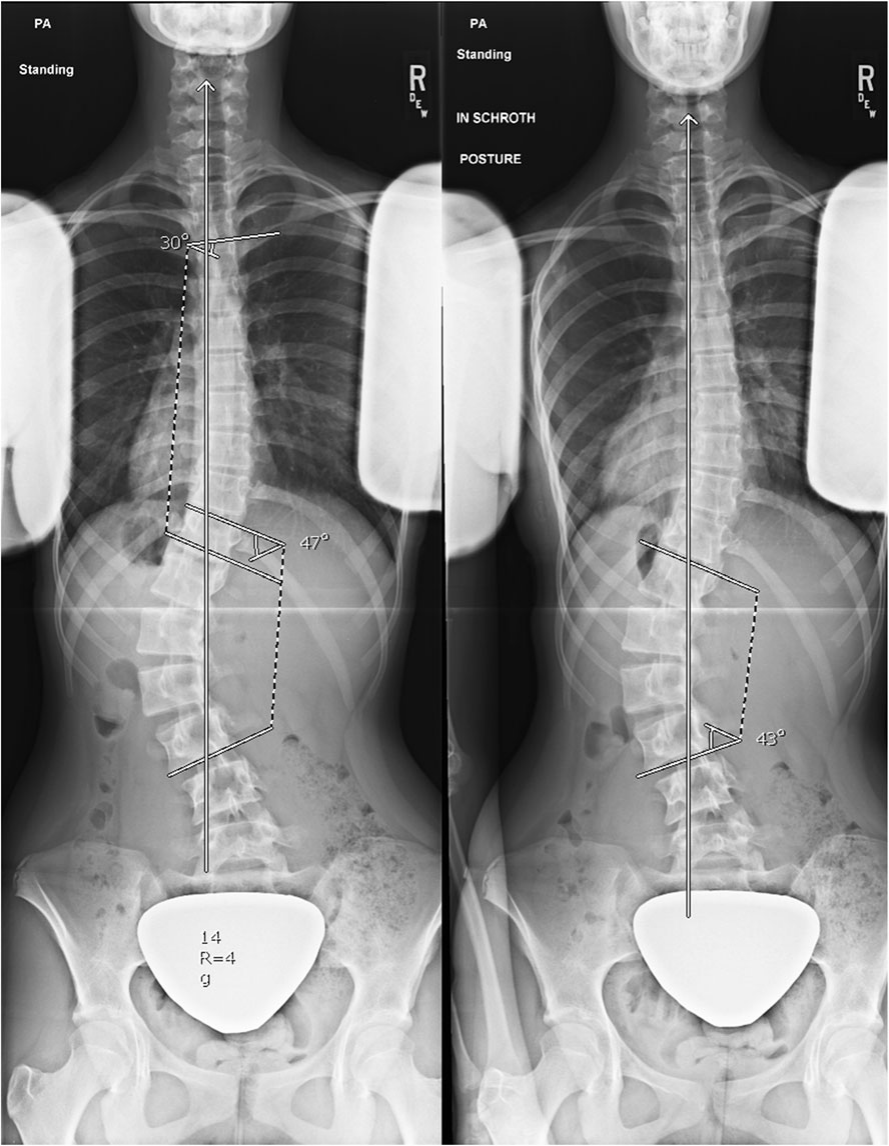
Initial exercise training. Relaxed standing (left) and corrective exercises (right)

**Fig. 5:**
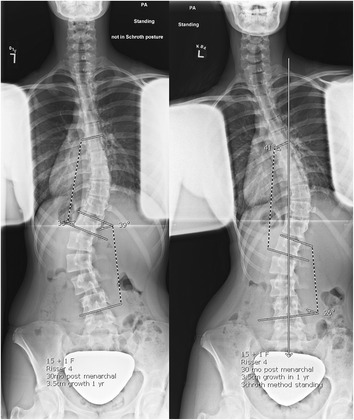
One year after exercises. Relaxed standing (left) and corrective exercises (right)

At 18 months, her lumbar and thoracic curves measured 47^o^ and 30^o^, respectively. Her Risser was 4. The brace was discontinued. Also at 18 months, immediately after her x-ray in the relaxed standing position, she performed her corrective exercises in standing with arms lowered for a second x-ray (Fig. 4). She performed the pelvis corrections for her curve type, auto-elongation, opening of her concavities, depressing her convexities, and shoulder counter-traction. Her lumbar and thoracic curves changed by less than 5^o^ and measured 43^o^ and 32^o^, respectively. At 30 months, one year after exercise instruction, her lumbar and thoracic curves measured 26^o^ and 41^o^, respectively. She later explained her improved x-ray, stating she had used corrective exercises during the x-ray (without being asked). Another x-ray was obtained in relaxed position during the same visit. Her lumbar and thoracic curves measured 39^o^ and 35^o^, respectively (Fig. 5). She was Risser 4 and had grown 1 cm the past year. The patient and parents reported home exercise compliance at 1–3 times a week the past year. The immediate effect of corrective exercises after a year of training was a 33 % improvement (13^o^) at the lumbar spine compared to only a 9 % (4^o^) improvement the previous year. The Cobb angle at the lumbar curve improved in the relaxed standing position by 8 ^o^ in the standing relaxed position compared to the 18 month visit. However, the Cobb angle at the thoracic curve worsened from 18 to 30 months (5° in the standing relaxed position and 9° with unsolicited correction).

## Conclusion

The patient’s thoracic curve worsened slightly from her 18 month visit to her 30 month visit although she approached skeletal maturity (Risser 4, 1 cm growth). She was treated as a 4C curve type (dominant lumbar curve with a nondominant thoracic curve), but the focus of the corrections were at the main curve (lumbar spine). It is difficult to say whether the corrective exercises had a negative effect on the nondominant thoracic curve, since clinically the patient appeared more balanced. Auto-corrrective exercises should improve alignment throughout the spine in three dimensions [4]. However, it can be challenging to improve each aspect of spinal deformity with multiple and even opposing body blocks. Perhaps a bigger focus should have been placed on the thoracic curve. The thoracic spine is stiffer than the lumbar spine due to the ribs attaching to the lumbar spine. Future studies should investigate whether other patients have experienced curve progression at the nondominant curves despite approaching skeletal maturity. Other Schroth studies only reported curve magnitude at the main curves [6, 7, 9] or did not report data specifically about the secondary curves [8].

Since patients are able to improve their Cobb angle for radiographs, we emphasize that patients should stand in their relaxed posture for x-rays at all visits to follow proper standardized radiographic procedures. However, it is possible that patients that utilize scoliosis-specific exercises perform their corrective exercises during radiographs which may result in overestimated exercise effects. However, one could also argue that radiographs displaying exercise corrections despite requesting that patients stand in a relaxed natural position can capture patients’ ability to hold a corrected position throughout their daily life. Future exercise studies should clarify instructions given to patients during radiographic acquisition. Also, with an increasing availability of EOS imaging which includes significantly lower radiation effects, [12] facilities could consider imaging patients in both their relaxed and corrected postures. EOS imaging may monitor the ability of patients to perform their corrective exercises, to understand the relative effect of various corrective instructions, and to determine the effect of the three-dimensional nature of the exercises (planes of maximum deformity or angles of rotation).

After initial training, corrective exercises during a standing x-ray did not significantly improve the Cobb angle for the major lumbar curve compared to the relaxed standing x-ray. However, a year after performing exercises, unsolicited corrective exercises resulted in a significantly improved Cobb angle compared to relaxed standing for the curve primarily targeted by the exercise program. Improved exercise ability and spinal flexibility may have contributed to the improved Cobb angle. Wearable technology is needed to better understand how much time patients are spending in their corrected versus relaxed postures.
